# Overt Infection with Chronic Bee Paralysis Virus (CBPV) in Two Honey Bee Colonies

**DOI:** 10.3390/vetsci7030142

**Published:** 2020-09-22

**Authors:** Julia Dittes, Marc O. Schäfer, Heike Aupperle-Lellbach, Christoph K. W. Mülling, Ilka U. Emmerich

**Affiliations:** 1 Centre for Applied Training and Learning, Faculty of Veterinary Medicine, Leipzig University, An den Tierkliniken 19, 04103 Leipzig, Germany; 2 Institute of Veterinary Anatomy, Histology and Embryology, Faculty of Veterinary Medicine, Leipzig University, An den Tierkliniken 43, 04103 Leipzig, Germany; c.muelling@vetmed.uni-leipzig.de; 3 Friedrich-Loeffler-Institut, Federal Research Institute for Animal Health, Südufer 10, 17493 Greifswald, Insel Riems, Germany; Marc.Schaefer@fli.de; 4 LABOKLIN GmbH & CO.KG, Labor für klinische Diagnostik, Steubenstraße 4, 97688 Bad Kissingen, Germany; aupperle@laboklin.de; 5 VETIDATA, Institute of Pharmacology, Pharmacy and Toxicology, Faculty of Veterinary Medicine, Leipzig University, An den Tierkliniken 39, 04103 Leipzig, Germany; emmerich@vetmed.uni-leipzig.de

**Keywords:** chronic bee paralysis virus, *Varroa* infestation control, nosemosis, hairless black syndrome

## Abstract

Chronic Bee Paralysis Virus (CBPV), a widespread honey bee RNA virus, causes massive worker bee losses, mostly in strong colonies. Two different syndromes, with paralysis, ataxia and flight incapacity on one hand and black hairless individuals with shortened abdomens on the other, can affect a colony simultaneously. This case report presents two *Apis mellifera carnica* colonies with symptoms of paralysis and hairless black syndrome in 2019. Via RT-PCR, a highly positive result for CBPV was detected in both samples. Further problems, such as a *Nosema* infection and *Varroa* infestation, were present in these colonies. Therapy methods were applied to colony 1 comprising queen replacement, shook swarm method and *Varroa* control, whereas colony 2 was asphyxiated after queen loss and colony weakening. After therapy, colony 1 was wintered without symptoms. Beekeeping and sanitary measures can save a CBPV-infected colony, while further complications result in total colony loss.

## 1. Introduction

The Western honey bee, *Apis mellifera* L., the most important pollinator in agriculture worldwide, is managed with the additional aim of honey production. Different pathogens are threatening these honey bee colonies. According to the collaborative German bee monitoring project with around 100 beekeepers involved, the most important diseases are varroosis, nosemosis and various bee viruses [1]. Nosemosis is a disease caused by the microsporidia *N. apis* and *N. ceranae*. They are obligate intracellular pathogens causing dysentery and diarrhea after parasitizing and damaging the epithelial cells of the midgut. In spring 2018, the honey bee colonies involved in the German monitoring programme were *Nosema*-positive in 44% of cases [2].

*Varroa*, a reddish-brown crab-shaped mite, is one of the most important reasons for winter losses and the main pathogen posing a risk to honey bee colonies. It weakens the bees by feeding from the bee’s fat bodies and harms the brood during their reproduction cycle in the combs [3]. In addition to being harmful itself, *Varroa* serves as a vector for various bee viruses, e.g., for the Deformed Wing Virus. In 2008, Celle et al. detected 1.4 × 10^4^ copies of Chronic Bee Paralysis Virus in *Varroa* mites and recognized the possibility of an infection of different hymenoptera like *Formica rufa* [4]. *Varroa*, then discussed as being a natural reservoir of CBPV, is rather unlikely as a vector, because there is no proven causal relation between Varroa infestation and CBPV in honey bee colonies. CBPV, the first bee virus ever described and isolated in 1963 [5], is a positive-sense, single-stranded RNA virus that is spread all over the world and mostlyaffects strong colonies with two different syndromes, often simultaneously seen in colonies—paralysis, trembling and crawling bees on one hand and hairless, black bees, with shortened abdomens on the other hand—both ending up in massive worker bee losses. Aristotle has already described the symptoms of the hairless black syndrome and called those bees “thieves” [6]. All casts of bees can be infected by the virus, but there seems to be a kind of behavioral protection for the queen bee [7], which might be the reason that often the queen and some workers remain in an affected colony.

The predominant neurologic symptoms, like the eponymous paralysis and ataxia, are caused by the neurotropism of the virus. Although the head comprises just 1/10 of the whole bee mass, half of the CBPV copies in an infected bee are located there, especially in neurons of the mushroom bodies and central complex, regions where movements are controlled [8]. Diseased bees are attacked by their hive mates, who cut off their hairs with their mandibles and cause the hairless black abdomens [9]. Ingesting the hairs of infected bees is one way of infection. Other possible infection routes are infection through feces or trophallaxis [4].

Almost identical symptoms are produced by Acute Bee Paralysis Virus, but bees die in between one and two days after infection. In contrast, chronically paralyzed bees live for several days in cage experiments [10]. In recent years, Chronic Bee Paralysis seems to be an emerging disease. Budge et al. [11] studied CBPV prevalence in England and Wales between 2007 and 2017. The number of reported cases increased from 1 case in 2007 to 45 cases in 2017 [11]. Other countries see rising numbers of CBPV-positive colonies in their monitoring, e.g., the US with 0.7% in 2010 and 16% in 2014 [12] or Italy with 5% in 2009 to 10% in 2010 [13]. The German bee monitoring also mentioned a notably high prevalence of CBPV in the investigated colonies in 2013 (35.8%) and 2014 (20.7%) [2].

This case report shows the course of two colonies in Germany suffering from an overt CBPV infection in 2019.

## 2. Case Description

### 2.1. Medical History, Appearance of the Hive and Environment

Both *Apis mellifera carnica* colonies are situated in Leipzig (51°20′36.5244″ N, 12°23′15.9792″ E), a city in the Federal Republic of Germany in a region with oceanic climate with continental influence.

**Figure 1:**
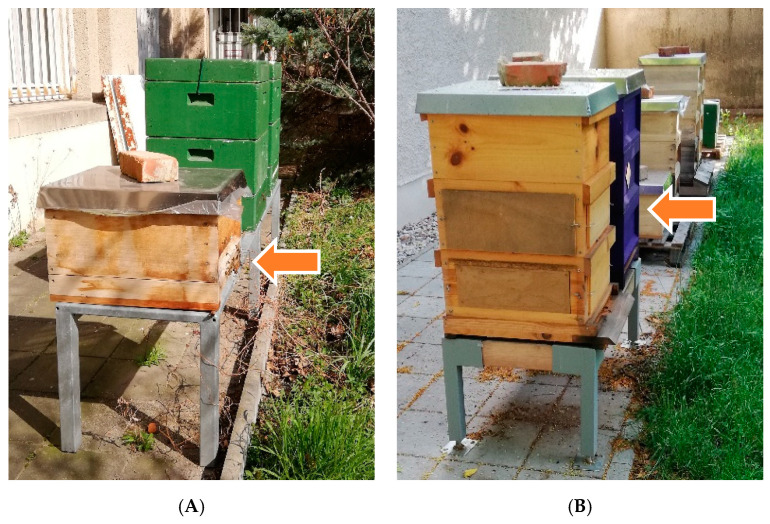
(**A**) Apiary 1 with colony 1 in a wooden Zander hive with one brood chamber in March 2020 (orange arrow), ©Julia Dittes. (**B**) Apiary 2 with colony 2 in a violet painted wooden Zander hive with two brood chambers and one honey super (second in the row) in June 2019 (orange arrow), ©Julia Dittes.

Colony 1 was situated next to two polystyrene hives on a 0.5-m-high metal stand in a wooden Zander hive. The hive consisted of one honey super and two brood chambers with ten frames each. It stood in an institute courtyard in an area with many chestnut, robinia and linden trees in the south of the city (Figure 1A). The entrance holes were oriented to the south.

Colony 2 was part of an apiary with six hives at a location a few hundred metres away from apiary 1. The wooden Zander hives stood in pairs on wooden pedestals or metal racks in the south of a small ancillary building with their entrance holes facing south as well. This hive also consisted of a honey super and two brood chambers with ten frames each (Figure 1B).

Both colonies were managed under the principles of good beekeeping practice and had been in a healthy status during the previous season.

*Varroa* infestation in both colonies was controlled by a combination of biotechnical methods and miticides. On 8 April 2019, the first frames without foundation and wire were put into the colonies for building drone combs. During the weekly health checks, drone brood was removed from the hive regularly and the building frames put back into the colony.

### 2.2. Examination of the Alighting Board and Observation of the Entrance Hole

**Figure 2:**
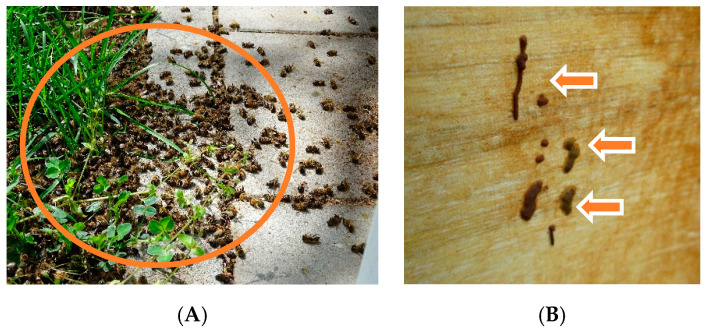
(**A**) Dead bees laying on the stone floor tiles and grass in front of the hive of colony 2 (orange circle), ©Julia Dittes. (**B**) Close-up of scattered brown feces found on the hive of colony 1 (orange arrows), ©Julia Dittes.

Observation of the entrance holes of both colonies in the beginning of May 2019 showed some isolated bees flying. Compared to the other hives at the apiaries, colony activity was significantly lower. Some dead bees lay on the alighting boards and a large quantity of dead bees was found in front of the hives (Figure 2A), which produced a certain putrid smell. Between those dead bees, some flightless crawling ones were spotted.

At the front of the first colony’s hive, additionally some scattered dark brown feces were visible (Figure 2B).

### 2.3. Clinical Examination of the Honey Bee Colonies and Observation of Living Bees

**Figure 3:**
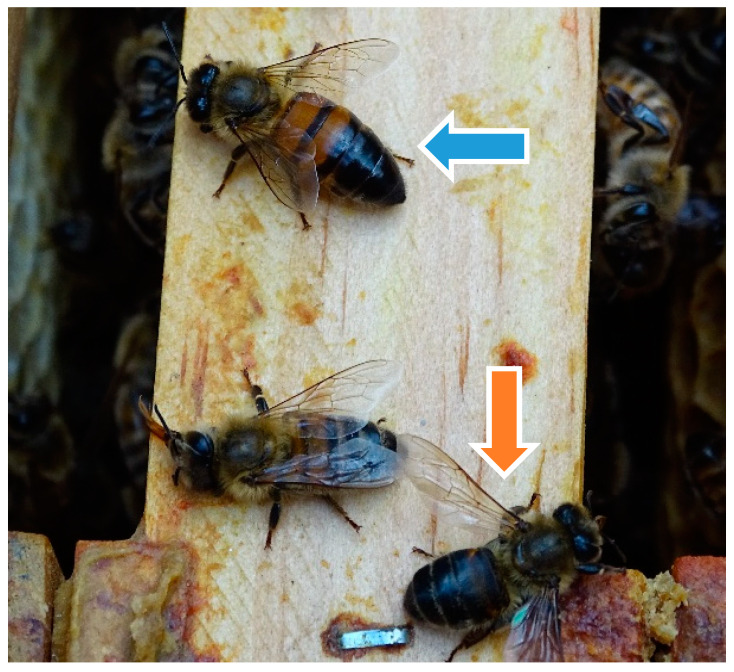
Clinical examination of colony 1: worker bee with hairless, “black” abdomen (blue arrow); worker bee with ant-like phenotype (orange arrow), ©Julia Dittes.

Clinical examination of colony 1 showed an empty honey super with undeveloped wax foundations and many bees with hairless, dark and shortened abdomens as well as some bees looking like ants in the brood chambers (Figure 3). The affected bees were trembling, atactic, with slow or no reaction when exposed to smoke, and held their wings at an unusual angle. Their movements were abnormal and erratic. Workers as well as drones showed clinical signs of paralysis (Supplementary Materials Videos S1 and S2). The nest consisted of five frames, one of them with drone brood, four with worker cells, and two regular drone brood frames. A large number of drones was seen in the colony.

The same clinical findings were observed in colony 2, with a brood nest out of six frames and two drone brood frames in the upper brood chamber. According to the Liebefeld method [14,15], with more than 7000 bees estimated, both colonies had nearly the same number of worker bees in the upper brood chamber, whereas the number of brood cells differed (approximately 14,200 in colony 2; 15,400 worker bee cells in colony 1).

**Figure 4:**
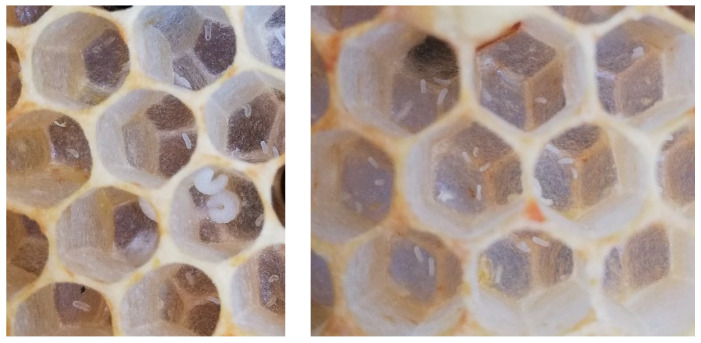
Close-ups of a drone brood comb of colony 2 with more than one egg or larvae per cell, ©Julia Dittes.

In contrast to colony 1, colony 2 started storing honey (approximately 4.1 kg) in the honey super. During the course of the disease, the dead queen of colony 2 was found in front of the hive on 10 May 2019. Later on, worker bees started laying eggs into the cells of colony 2 (Figure 4).

From these unfertilized eggs, only drones developed, and the colony weakened progressively.

#### Examination of Dead Bees

Samples of dead bees were examined using the methods described in Dittes et al., 2020 [16]. The bees of both colonies showed the same symptoms as previously seen in the colony: Some bees were about 3 to 4 mm smaller than common, most of the bees had an extended proboscis. The abdomens were hairless, black, greasy shining, shortened and in some individuals bloated. Under pressure on the abdomen a light brown fluid leaked from the gut.

The queen of colony 2, showed the same problems, as well as drones of both colonies, so that all casts of bees were affected by the disease.

### 2.4. Laboratory Diagnosis

#### 2.4.1. PCR-Diagnosis of CBPV and Further Viruses

RT-PCR was performed for both colonies at the end of May 2019 at the Federal Research Institute for Animal Health (FLI, Greifswald–Isle of Riems). After homogenizing ten bees from each sample with the gentleMACS^TM^ Dissociator, total RNA was purified from 150 µL of clarified bee homogenate using the RNeasy Mini Kit (Qiagen, Venlo, NL, USA). A one-step real-time RT-PCR was subsequently performed in duplicate using the AgPathID^TM^ One-Step RT-PCR Kit (Applied Biosystems^TM^, Waltham, MA, USA) in a 96-well reaction plate [17]. Detailed information of the methods used can be found in Dittes et al., 2020 [16]. The results are expressed as the mean of the two replicates for each reaction.

Both samples were positive for CBPV showing a threshold cycle (Ct) below 20 for each amplification. Furthermore, titres of Deformed Wing Virus (DWV) and Black Queen Cell Virus (BQCV) were detected in the samples of both colonies without causing clinical findings. Acute Bee Paralysis Virus (ABPV) was not or barely detected (Table 1).

#### 2.4.2. Diagnostics of Further Diseases

##### Monitoring the Varroa Infestation

There are different methods to estimate the mite load [16]. The number of mites in this case was estimated from the natural mite fall, the number of mites per day falling naturally from the bees down to the bottom board. An oil-coated paper towel was placed on a drawer under the wire-mesh floor for 2 to 3 days and the mites, naturally fallen from the bees to the floor, were counted in the debris. Female *Varroa* mites are from 1.2 to 1.7 mm, crab-shaped and reddish brown and can be seen with the naked eye [18]. In Table 2, the control plan and the natural mite fall with a merely low infestation rate are shown for colony 1.

##### Detection of Nosemosis

**Figure 5:**
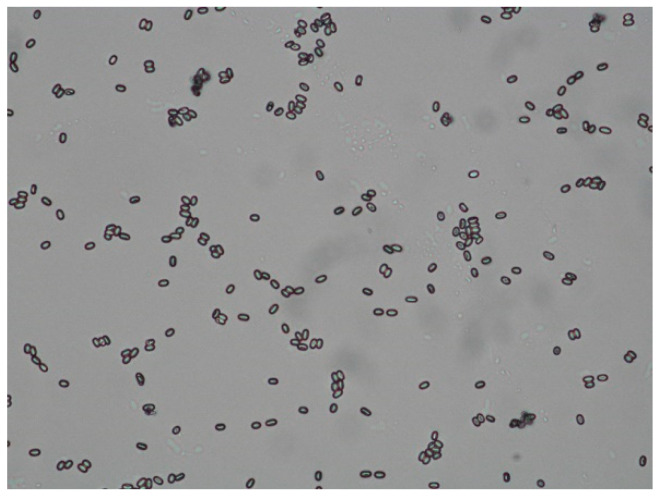
Microscopic image of oval-shaped *Nosema*-spores. (unstained, 400x) Differentiation of the species is not possible under the microscope. ©Heike Aupperle-Lellbach.

A microscopic estimation of the number of spores was carried out using the scheme described by Ritter in 1996 [19]. Colony 1 had a moderate infestation (between 20 and 100 spores per visual field), Colony 2 was only slightly affected (less than 20 spores per visual field) (Figure 5). For further differentiation of the species *N. apis* and *N. ceranae,* a PCR would be required, but was not performed in this case.

### 2.5. List of Medical Issues and Diagnoses

Table 3 lists the main problems of the colonies. In sum, an overt infection with Chronic Bee Paralysis Virus was diagnosed in both colonies. In addition, a co-infection with *Nosema* was proven with different infestation grades in the colonies.

Treatment methods were only applied to colony 1. After queen loss, a surplus of drones and laying worker bees, the prognosis was too bad for colony 2.

### 2.6. Therapeutic Measures and Outcome

Due to the bad prognosis, colony 2 was asphyxiated with sulphur on 17 June 2019. 

For therapy of colony 1, measures such as re-queening, shook swarm method and *Varroa* infestation control were applied. Further information on those methods can be found in Dittes et al. 2020 [16]. Table 4 summarizes the therapeutic measures and further development of colony 1.

**Figure 6:**
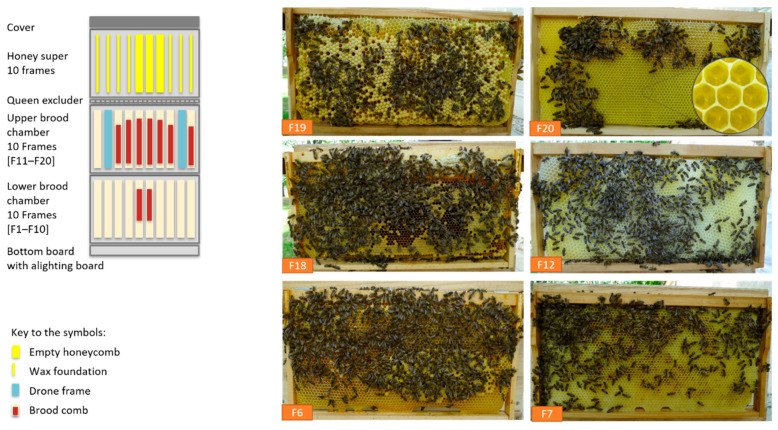
Development of the rehabilitated colony 1 after wintering/hibernation on 28 April 2020 during apple blossom (brood on 9 combs [Zander 477 × 220 mm], honey super empty), F19: capped drone brood, F20 + F7: brood-comb with eggs, F18 + F6: brood-comb with mostly covered worker brood, F12: build up drone brood frame ©Ilka Emmerich.

No symptoms of paralysis were shown by the bees during the weekly check-ups in 2020 (Video S3). CBPV was not detected via RT-PCR any longer. The vital colony (Figure 6) started honey production in the following weeks. In total, about 36 kg of honey was harvested from colony 1 in 2020 in an apiary averaging 42 kg of honey per colony.

## 3. Discussion

Paralysis of honey bees was systematically studied for the first time by Burnside [20] nearly 100 years ago in 1933. Performing many cage experiments spraying, injecting and feeding healthy bees with extract from sick bees, he observed bees attacking their hive mates [20]. After further infection experiments in 1945, Burnside drew the conclusion that a filtrable virus is the cause of the disease [21]. However, it took another 20 years until Bailey first isolated Chronic Bee Paralysis Virus [5], a virus that causes neurologic symptoms. Clinical findings are trembling, crawling, circling bees and flightless bees without orientation, because the virus affects neurons of higher-order integration centres, optic and antennal lobes, which are involved in locomotion control, learning and orientation behavior [18]. At the same time, phenotype changes in bees are seen: black hairless shiny abdomens and smaller bees with shortened abdomens. In the beginning, CBPV was characterized by two distinct syndromes, the paralysis form and the hairless black syndrome, but both can occur in colonies at the same time, as was observed in the two described ones. All symptoms may lead to a high mortality within a few days, with massive worker bee losses.

Acute Bee Paralysis Virus, which produces the same symptoms but leads to a faster death of affected bees, could be excluded through differential diagnosis via PCR, where it was not or just marginally detected.

In formerly strong colonies just the queen and a few workers remain. Amiri et al. [7] compared the queen’s and the worker bee’s susceptibility. Infection experiments for both workers and queens resulted in the same symptoms after 6 days and a 100% mortality after 14 days. There seem to be behavioral strategies to protect the queen, e.g., only healthy bees feeding her [7]. The observations in this case confirm that for colony 1. The queen of colony 2 died and was found in front of the hive. However, it is not possible to clearly determine the cause of the queen’s death. She could have been affected by CBPV, too, but she also could have been harmed while handling the colony or was to be replaced by a new queen, because of failures detected by the workers. Replacement by supersedure, which is done with old queens, can be nearly ruled out, because it was a young queen raised in 2018, one year prior. Normally, the colony would have reared a new queen. However, instead, laying worker bees started ovipositure in a disordered way in the absence of the queen’s pheromones. From these unfertilized eggs, only drones developed, and with a declining number of working honey bees, the colony weakened and could not be rehabilitated. Asphyxiating with sulfur is the standard method to end a colony’s life, as was done to colony 2.

CBPV often persists as a covert infection in honey bee colonies, detectable via PCR, without causing obvious symptoms, clinical findings and problems within the colony. Different factors favour the change into an overt infection. The interaction between these factors is not understood in detail yet, but periods of bad weather or a lack of nectar and starving are discussed. Taking a look at the weather in May 2019 in Germany, the temperatures were about 1.2 °C colder than normal, especially during the first half [22]. Cold and rainy conditions outside make the bees stay in the hive, intensifying their direct contact and may facilitate the spread of the virus. Direct contact plays a predominant role in transmission during disease outbreak [8].

Infected bees suffer from nibbling attacks of their hive mates, cutting their hairs off. This leads to the black hairless shiny abdomens of the attacked ones and the ingestion of bee hairs by the attackers. Rinderer et al. investigated the effect of hairs removed from the bodies of infected honey bees in infection experiments and saw that bees fed with virus and infected hairs showed a significantly higher mortality than bees fed only with one of both [9].

A high density of bees in the hive also increases the amount of and the contact with feces, another possible way of transmission. The virus load in bee excreta is as high as in the heads of symptomatic bees (10^10^ CBPV RNA copies) [23]. In the described colonies a co-infection with *Nosema* was detected. These microsporidia cause dysentery and diarrhea through damage of the midgut epithelial cells, which results in more feces in the hive, especially under the weather conditions described above. Toplak et al. investigated the effect of a co-infection of CBPV and *Nosema ceranae* in bees under experimental conditions. Bees infected with *Nosema* showed an increased replication ability for CBPV [24]. The damage to the midgut epithelial cells and a suppression of the immune response of the honey bees increase the virulence of the viral pathogens. The co-infection may have had a synergistic effect on the CBPV outbreak in the described cases. While the species were not differentiated via PCR, according to the German bee monitoring, the dominant species was *Nosema ceranae* in more than 96% of the *Nosema*-positive samples in 2018 [1,2], so it is the most likely species here as well.

Furthermore, the *Varroa* mite was discussed to be a vector and natural reservoir for CBPV. Investigations into mites showed a titre of 1.4 × 10^4^ particles per mite, which is quite low. However, minus strand RNA was detected, which is indicative of virus replication. *Varroa* was and is present in the described colonies and also acts as a general weakening factor for honey bee colonies, therefore an intense *Varroa* infestation control has to be done.

There are no specific drugs or therapy measures against viral diseases, so a therapy has to rely on sanitary measures and basics of good bee keeping. Bailey described in 1965 that re-queening was a helpful measure in naturally CBPV-infected colonies [5]. In surviving colony 1, re-queening was the first step to rehabilitation, followed by the shook swarm method. Artificial swarming is often used to decrease the load of a pathogen in the bee hive, e.g., of American foulbrood. The bees got a new hive with new and clean combs. In the same step, a brood-less period was induced, allowing for a *Varroa* treatment by spraying oxalic acid. With the combination of these measures, a total recovery of colony 1 was possible. In colony 2, the intervention came too late to save it.

## 4. Conclusions

If diagnosed in time, an overt CBPV infection can be treated successfully with bee keeping and sanitary measures. There is a good prognosis for the affected colony. In case of further complications, the infection can result in a total colony loss. A survey about therapy measures and outcome among beekeepers with confirmed CBPV-positive bee colonies during recent years should gather information on successful strategies to be applied to affected colonies in the future.

It remains to be seen how CBPV will spread in the next years, and whether Germany will experience a similar growth to England and Wales. Furthermore, research about the specific factors that lead to an outbreak is required in order to develop strategies for preventing overt infections in colonies.

## Figures and Tables

**Table 1:** Virus titres detected in two honey bee colonies in May 2019.

Virus	Titres for Colony 1	Titres for Colony 2
CBPV	++++ (Ct = 13.45)	++++ (Ct = 13.08)
DWV-B	+++ (Ct = 25.31)	+++ (Ct = 23.10)
BQCV	++++ (Ct = 18.27)	+++ (Ct = 27.56)
ABPV	+ (Ct = 34.65)	- (N/A)

Legend: CBPV = Chronic bee paralysis virus, DWV = Deformed wing virus, BQCV = Black Queen Cell Virus, ABPV = Acute bee paralysis virus, Ct = threshold cycle; classification of Ct: ++++, Ct < 20; +++, 20 ≤ Ct < 28; ++, 28 ≤ Ct ≤ 32; +, 32 < Ct ≤ 35; (+), Ct > 35; N/A, no evidence.

**Table 2:** *Varroa* screening plan in 2019 by debris examination for the surviving colony 1.

Plastic Drawer		Natural Mite Fall
Into the Hive	Out of the Hive	
23 July	25 July	0m/2d → 0m/d
16 August	19 August	0m/3d → 0m/d
11 September	13 September	6m/2d → 3m/d
25 November	28 November	0m/3d → 0m/d

Legend: m = mite, d = day.

**Table 3:** List of medical issues in two honey bee colonies infected with Chronic Bee Paralysis Virus (CBPV) in 2019.

Colony 1	Colony 2
for both colonies: - hairless, black bees - smaller bees with shortened abdomens - atactic, crawling and trembling bees - bees with bloated abdomens - large number of dead bees in front of the hives - positive PCR result for CBPV (Colony 1: ++++ (Ct = 13.45), Colony 2: ++++ (Ct = 13.08))	
moderate infestation of Nosema spores	slight infestation of Nosema spores
	loss of the queen
	laying worker bees and large number of drones
other viruses (without symptoms): DWV-B, BQCV, ABPV (exact Cts in Table 1)	other viruses (without symptoms): DWV-B, BQCV, ABPV (exact Cts in Table 1)

Legend: DWV = Deformed Wing Virus, BQCV = Black Queen Cell Virus, ABPV = Acute Bee Paralysis Virus, Ct = threshold cycle

**Table 4:** Therapeutic measures and outcome of colony 1 after diagnosis of an overt CBPV infection.

Date	Applied Measures/Methods and Development of Colony 1
29 May 2019	removal of the old queen from the hive, bees start raising a new queen
06 June 2019	destruction of all of the queen cells, insertion of a queen cage with the new queen (Nr. 71, marked green) and some food, gentle acclimatization while workers gnaw through the food
18 June 2019	hive check: many eggs and young brood in the brood combs, queen Nr. 71 with a larger abdomen
19 June 2019	shook swarm method: migration to a new hive body with new pathogen free frames and wax foundations, protection of the queen in a queen cage, bees shaken off in front of the new hive onto a base to enter the new hive by themselves.
21 June 2019	treatment with 5.7% Oxalic acid dihydrate (OXUVAR^®^ 5.7%, Andermatt Biovet GmbH, Lörrach, BW, D)) diluted to a 3.5% solution by spraying; Feeding with sugar syrup (2 kg sugar)
25 June 2019	decreased symptoms of trembling and paralysis or black abdomens
28 June 2019	feeding with sugar syrup (2 kg sugar)
1 July 2019	feeding with sugar syrup (2 kg sugar)
17 July 2019	feeding with sugar syrup (2 kg sugar)
21 August 2019	feeding with sugar syrup (2 kg sugar)
16 September 2019	late summer treatment with formic acid (Ameisensäure 60% ad us. vet., Serumwerk Bernburg AG, Bernburg, ST, D) after the last honey harvest: evaporation of 140 mL of formic acid in the hive using a Liebig Dispenser; observed–mite load (Table 1): about 3 mites per day = medium infestation rate [18]
October 2019–April 2020	overwintering of the colony with only one brood chamber, no application of a winter treatment with oxalic acid because of a low mite infestation rate in November (Table 1)
12 April 2020	successful hibernation; enlargement of the hive with a second brood chamber.
28 April 2020	brood nest out of 9 brood combs during apple blossom

## Supplementary Materials

The following are available online at https://www.mdpi.com/2306-7381/7/3/142/s1, Video S1: trembling and ataxia, hairless, black bees in colony 1_May 2019; Video S2: trembling_paralysis_hairless black bees in colony 2_May 2019; Video S3: normal behaviour of colony 1 in May 2020.
